# *Cancer PathogenesisandTherapy*: A new journal, a new inspiration

**DOI:** 10.1016/j.cpt.2022.10.001

**Published:** 2022-10-08

**Authors:** Yuankai Shi, David James Kerr

**Affiliations:** Department of Medical Oncology, National Cancer Center/National Clinical Research Center for Cancer/Cancer Hospital, Chinese Academy of Medical Sciences & Peking Union Medical College, Beijing Key Laboratory of Clinical Study on Anticancer Molecular Targeted Drugs, Beijing 100021, China; Radcliffe Department of Medicine, University of Oxford, Oxford, OX1 4DJ, UK

**Keywords:** Medical journal, Oncology, Publication

Dear readers:

It is our great pleasure to introduce *Cancer Pathogenesis* *and* *Therapy (CPT)*, a journal officially launched and supported by the Chinese Medical Association (CMA) and the only international English-language journal in the field of oncology published by the CMA. As the Editor-in-Chief appointed by the CMA, on behalf of the editorial team, we would like to extend a warm welcome to the readership of *CPT*. We would also like to take this opportunity to thank our authors, editors, and reviewers, all of whom contributed tremendously to the successful launch of this journal.

In the wave of globalization and information technology development, an increasing number of Chinese scholars have made important and prominent contributions to research advances at a worldwide level. However, the number of English-language journals in China is limited, particularly in the field of oncology. Accordingly, many excellent research results are not publicly available because of the lack of convenient and suitable platforms for publication. In October 2019, the China Association for Science and Technology decided to launch a new high-quality English-language journal in the field of oncology as part of the High Notch New Journal Project in its Excellence Action Plan. Thus, *CPT* was established to undertake this vital mission.

*CPT* is organized and supervised by the Chinese Medical Association Publishing House, the largest medical publishing organization in China. The journal is dedicated to publishing innovative and significant results in basic research, clinical research, cancer prevention, and public health policy, including novel anticancer drugs, with a particular focus on clinical trials related to intelligent tumor diagnosis and treatment and translational research. *CPT* is committed to building a world-class academic communication platform that will rapidly publish high-quality scientific research results in all areas of oncology for a broader audience. We will also host a series of academic conferences and workshops, particularly for young physicians and researchers worldwide. Moreover, *CPT* will release important guidelines and expert consensus to provide widely available and accessible authoritative information in specialized fields.

*CPT* is committed to upholding the highest ethical standards in medical publishing. To ensure the highest standards of scientific quality, integrity, and professionalism, we are fortunate to have many distinguished international experts serving on the Editorial Board, which consists of members of the international research and clinical community of oncology and relevant specialties, including basic cancer research, clinical cancer research, oncological pharmacology, oncologic imaging, cancer genomics, cancer informatics, artificial intelligence application, cancer prevention, public health policy, and translational medicine. Editorial Board members were recommended based on their academic success and expertise in their respective fields. We appreciate the academician of the Chinese Academy of Engineering, Guowei Sang, as Honorary Editor-in-Chief; and Professors Jianxiang Wang and so on from China, along with Professors Karel Pacak (USA) and so on for serving as Associate Editors. Over 40 distinguished oncologists and researchers, including Professors Changhao Wu (UK), and many others, were invited to serve as Editorial Board members. We firmly believe that this outstanding international team will bring *CPT* to a prominent place in the field of oncology.

*CPT* is devoted to publishing high-quality Original Studies, Reviews, Meta-analysis, Research Highlights, Editorials, Guidelines and Consensus, Case Reports, Clinical Images, Study Protocols, Letters, and other article types. The journal is in a position to be the bearer of and window to innovative findings and initiations in a timely manner. This will play an important role in the development of promising fields of oncology.

The predecessors of the medical profession in China consistently worked hard to promote the development and propagation of medical science. In February 1915, the CMA was established in Shanghai by 21 Chinese physicians, including Dr. Lien-teh Wu and Dr. Fu-ching Yen. The first version of the *National Medical Journal of China*, with both Chinese and English editions, was published the following November. The first issue declared the nascent organization's aim to unite medical professionals, uphold medical ethics and the rights of doctors, disseminate medical knowledge, advocate for social justice, and liaise with international medical circles. Since then, Chinese medical professionals have established their own organizations and have played an independent role in the development of medicine. This year, as part of the 108 th anniversary celebrations, we launched *CPT* as a new journal, which we believe is highly anticipated by the oncology community worldwide, and it brings us great pleasure to write this inaugural message.

## Conflicts of interest

The authors declares that they have no known competing financial interests or personal relationships that could have appeared to influence the work reported in this paper.

